# Trichothecene Genotypes of the *Fusarium graminearum* Species Complex Isolated from Brazilian Wheat Grains by Conventional and Quantitative PCR

**DOI:** 10.3389/fmicb.2016.00246

**Published:** 2016-03-01

**Authors:** Sabina M. Tralamazza, Raquel Braghini, Benedito Corrêa

**Affiliations:** Laboratory of Mycotoxins and Toxigenic Fungi, Institute of Biomedical Sciences, University of São PauloSão Paulo, Brazil

**Keywords:** genotype, trichothecenes, wheat, *Fusarium*, qPCR, deoxynivalenol

## Abstract

We compared two well-established methods, fungal isolation followed by conventional PCR and DNA analysis by quantitative PCR (qPCR), to define trichothecene genotypes in Brazilian wheat grains from different locations. For this purpose, after fungal isolation from 75 wheat samples, 100 isolates of the *Fusarium graminearum* species complex (FGSC) were genotyped by PCR to establish their trichothecene profile. For profiling by qPCR, DNA was extracted from the wheat samples and analyzed. The methods provided similar and divergent results. The FGSC isolates were classified as NIV (55%), 15-ADON (43%), and 3-ADON (2%). Analysis by qPCR showed 100% contamination with 15-ADON strains in all wheat samples, 80% contamination with the NIV genotype, and only 33.3% contamination with 3-ADON strains. Further analysis revealed that 96% of all quantified DNA was attributed to the 15-ADON profile, while 3.4% was attributed to NIV and only 0.06% to 3-ADON. A positive correlation was observed between 15-ADON genotype DNA concentration and deoxynivalenol (DON) content in the wheat samples. The high frequency of fungi, DNA levels and positive correlation with DON strongly indicate that 15-ADON producers are the main trichothecene genotype in Brazilian wheat grains. Surprisingly, although many isolates (55%) carried the NIV genotype and this genotype was identified in 80% of the wheat samples, only 3.4% of fungal DNA was in fact from NIV producers. Although, our findings showed that each method provided a different perspective about the trichothecene profile, DNA analysis by qPCR gave us new insight about fungal contamination levels in Brazilian wheat grains. Nevertheless, both techniques should be used to obtain more robust results.

## Introduction

The continuous increase in wheat production in Brazil (USDA, 2014) and recent mycotoxin regulations (ANVISA, 2011) indicate the urgent need for more studies about fungal diversity and mycotoxin profiles found in these grains to ensure a good-quality and safe product for human and animal consumption. Members of the *Fusarium graminearum* species complex (FGSC) are the main fungal agents associated with Fusarium head blight (FHB) in wheat and other cereal crops, a disease that causes severe grain losses for the industry every year (Goswami and Kistler, 2004; Osborne and Stein, 2007). In addition to causing FHB, FGSC produce type B trichothecenes and zearalenone, mycotoxins that pose hazardous health risks to humans and animals. The consumption of trichothecenes causes vomiting, feed refusal, anorexia and weight loss (Smith et al., 1997; Pestka and Smolinski, 2005), while zearalenone ingestion can induce significant changes in reproductive organs and fertility loss in animals and humans (Zinedine et al., 2007).

Type B trichothecenes synthesized by FGSC vary worldwide and their profile is normally divided into three categories: (1) nivalenol (NIV) and its acetylated form (4-ANIV); (2) deoxynivalenol (DON) and 15-acetyldeoxynivalenol (15-ADON); (3) DON and 3-acetyldeoxynivalenol (3-ADON) (Ward et al., 2002; Desjardins, 2006). Although these toxins are included in the same group, they may differ in terms of aggressiveness and toxicity (Lee et al., 2009; Puri and Zhong, 2010; Umpiérrez-Failache et al., 2013). In view of the possibility of different profiles, it is important to characterize potential mycotoxins in cereals and to analyze shifts in the toxigenic profile of fungal populations.

Trichothecene genotyping provides a rapid method to predict trichothecene production by *Fusarium* species. Current knowledge of *TRI* genes involved in the trichothecene biosynthetic pathway permitted to design specific primers for the identification of trichothecene genotypes. Primers based on the *TRI3* and *TRI12* genes were developed to differentiate 3-ADON, 15-ADON and NIV genotypes (Ward et al., 2002), and those based on the *TRI13* and *TRI7* genes to define DON and NIV genotypes (Chandler et al., 2003).

Most studies exclusively focus on genotyping strains isolated from wheat by PCR. Although less expensive, the technique is a qualitative analysis and requires a prior step, i.e., fungal isolation. On the other hand, qPCR permits qualitative and quantitative analysis and, more importantly, can be used to evaluate the genotype profile directly in the fungal substrate; however, the method is more expensive and requires a higher technical knowledge. Therefore, the objective of this study was to investigate the trichothecene profile of wheat grains using two methods, fungal isolation and conventional PCR and qPCR, in order to provide a more robust analysis of the trichothecene profile of the *Fusarium* population in Brazilian wheat.

## Materials and Methods

### Wheat Samples

The wheat samples used in the study were previously analyzed for mycobiota and DON content (Tralamazza et al., 2016). Of the 150 samples collected in that study, 75 were randomly chosen for the present study. The freshly harvested wheat grains were collected in three different states (25 samples/region) in Brazil. The regions were chosen due to their importance for the wheat industry. At present, the states of Parana and Rio Grande do Sul are responsible for more than 90% of the total wheat production in Brazil (CONAB, 2015). Harvest occurred between September and November 2012 in Novo Itacolomi (Parana State, PR), Passo Fundo (Rio Grande do Sul State, RS), and Capao Bonito (São Paulo State, SP). Grains were collected 3–6 days after harvest. Samples of approximately 1 kg each were collected and stored at 4°C for immediate analysis.

### Trichothecene Genotype Identification by Conventional PCR

#### Fungal Isolation

As mentioned earlier, the wheat samples and subsequent fungal isolates were part of a previous work. For the present study, 100 FGSC strains were used. These strains were directly isolated from the 75 wheat samples used in the qPCR genotype investigation.

A small percentage (<1%) of *Fusarium* trichothecene producers from other *Fusarium* complex were found during the mycobiota study but none were isolated from the 75 samples used in the present study. Thus, for this study we only worked with fungal samples from the FGSC.

Briefly, subsamples (100 g) of the wheat grains were disinfected with commercial sodium hypochlorite solution (1%) for 1 min and washed two times with distilled water. Subsamples (100 grains) were transferred to PDA plates (10 grains/plate) and incubated for 5 days at 25°C. For species identification, DNA was extracted as described in “Fungal DNA Extraction for Genotype Identification” and the elongation factor (EF-1α) gene was sequenced using the EF-1/EF-2 primers (O’Donnell et al., 1998). All amplification reactions were carried out in a volume of 25 μl containing 1x PCR buffer, 0.3 mM of each primer, 2.5 mM MgCl_2_, 0.2 mM dNTPs, 0.04 U/μl Taq DNA polymerase (Invitrogen, Carlsbad, CA, USA), and 100 ng template DNA. The PCR conditions were initial denaturation at 94°C (5 min), followed by 35 cycles at 95°C (30 s), 56°C (30 s) and 72°C (1 min), and a final extension step of 7 min at 72°C. After DNA purification (Illustra ExoProStar, GE), sequencing was performed in an ABI 3730 DNA Analyzer (Applied Biosystems, Foster City, CA, USA) using the BigDye Terminator v3.1. kit (Applied Biosystems) according to manufacturer instructions.

#### Fungal DNA Extraction for Genotype Identification

Monosporic strains were cultured for 5 days at 25°C on yeast extract sucrose (YES) agar. After growth, mycelia were scrapped off and DNA was extracted using the Easy-DNA kit (Invitrogen) according to manufacturer instructions.

#### PCR

Primers Tri13-F/Tri13DON-R were used for analysis of the DON profile and primers Tri13NIV-F/Tri13-R for NIV (Chandler et al., 2003). Multiplex PCR was performed to determine NIV, 3-ADON and 15-ADON genotypes using primers 12CON/12NF/12-15F/13-3F (Ward et al., 2002). Amplification was carried out in a Veriti Thermal Cycler (Applied Biosystems) using the following cycle parameters: 95°C (1 min), 25 cycles at 95°C (30 s), 52°C (30 s) and 72°C (30 s), and a final extension at 72°C (7 min). The amplification reactions was carried out in a volume of 25 μl containing 1x PCR buffer, 2.5 mM MgCl_2_, 0.3 dNTPs, 0.56 mM of each primer, 0.04 U/μl of Taq DNA polymerase (Invitrogen), and 100 ng template DNA.

### Trichothecene Genotype Identification by Quantitative PCR

#### DNA Extraction

A 50-mg aliquot of a 100-g ground wheat sample was transferred to a microtube with a 3-mm steel pearl and shaken for 3 min (50 rpm) on a TissueLyser LT (Qiagen, Venlo, The Netherland). Next, DNA was extracted using the Easy-DNA kit (Invitrogen) according to manufacturer instructions. Fungal isolates with defined trichothecene genotypes were cultured in YES agar medium for 5 days at 25°C and used for the construction of efficiency and standard curves. Mycelia were scrapped from the medium and DNA was extracted using the Easy-DNA kit (Invitrogen) according to manufacturer instructions. Fungal and wheat DNA concentrations were determined in a Nanodrop 2000 UV-VIS spectrophotometer (Thermo Fisher, Waltham, MA, USA).

#### Validation of the Method

Quantitative PCR analysis was carried out according to Nielsen et al. (2012). Briefly, 5-point calibration curves were constructed for the fungal isolates of each genotype (3-ADON, 15-ADON, and NIV) using defined quantities of DNA. PCR efficiency was calculated from the slope of the linear relationship between the log10 values of DNA quantity and the cycle number (E = 10^(-1/slope)^-1). Target genes were amplified using the trichothecene genotype-specific primers (Nielsen et al., 2012), and primers for plant elongation factor (TEF1-α) (Nicolaisen et al., 2009) were used to determine DNA yield and possible nucleic acid degradation.

#### Quantification of Trichothecene Genotype DNA

The qPCR conditions and cycle protocol described by Nielsen et al. (2012) were used. The qPCR assays were carried out in a StepOnePlus Real-Time PCR System (Applied Biosystems). For the determination of genotype DNA concentration, the cycle threshold (Ct) of each sample was compared to the standard curve of the fungal isolate of each specific genotype. To obtain the final value, genotype DNA was normalized to the plant elongation factor, resulting in pg fungal DNA per μg plant DNA (Nicolaisen et al., 2009).

### Deoxynivalenol Analysis

#### Materials and Reagents

Mycotoxin standards (DON and deepoxydeoxynivalenol) were purchased from Sigma- Aldrich (São Paulo, Brazil). Acetonitrile, methanol and ammonium acetate were purchased from J. T. Baker (São Paulo, Brazil). Ultrapure water was obtained with a Milli-Q-System from Merck Millipore (Bedford, MA, USA).

#### Extraction of Deoxynivalenol

Three gram of wheat grains was ground and homogenized in 24 ml of a methanol/water solution (70: 30, v/v) and shaken for 20 min. The mixture was filtered through a Whatman No. 4 filter (18 cm). Prior to liquid chromatography-mass spectroscopy (LC-MS/MS) analysis, a 40-μl aliquot was transferred to a vial, mixed with 955 μl methanol/water (50: 50, v/v), and 5 μl of the internal standards previously diluted in methanol/water (50: 50, v/v) was added.

#### Chromatographic Conditions

The content of DON had been determined in a previous study (Tralamazza et al., 2016). DON was analyzed in an Agilent 1200 HPLC System (Agilent Technologies, Santa Clara, CA, USA) equipped with an API5000 triple quadruple mass spectrometer with an electrospray source (AB Sciex, Concord, ON, Canada). The LC column was a C8 Zorbax-XDB, 200 × 4.6 mm, 3 μm (Agilent Technologies) equipped with a C8 pre-column cartridge. For the mobile phase, methanol/water (60:40, v/v) containing 0.05 M ammonium acetate was used in an isocratic procedure at a flow rate of 1 ml/min. The column temperature was 35°C and an injection volume of 5 μl was used. The MS source-dependent parameters were: curtain gas 30 psi (240 kPa of maximum 99.5% nitrogen), dry gas (GS1) 50 psi (380 kPa of zero grade air), dry gas (GS2) 20 psi (105 kPa of zero grade air), collision-activated dissociation gas 12 (arbitrary unit), source temperature 360°C, and ion spray voltage 5200 V. Detection was performed in the negative ion electrospray mode using multiple reaction monitoring. The retention time was 1.38 min. The declustering potential was set at -55 V, the collision energy at -30 eV, and the cell exit potential at -20V.

### Statistical Analysis

Statistical analysis was performed using the GraphPad Prism software (GraphPad, 2014, v. 6.05). The Kruskal–Wallis test and Pearson’s correlation test were used. A *p*-value < 0.05 was considered statistically significant.

## Results

### Trichothecene Genotype Identification by Conventional PCR

All three genotypes were identified in the fungal isolates. However, wide variation in the frequency of the profiles was found. As can be seen in **Table 1**, 55% of the isolated fungi carried the NIV genotype, 43% the 15-ADON genotype, and only 2% the 3-ADON genotype. Mycobiota analysis of the wheat samples revealed that the 15-ADON genotype was attributed to *Fusarium graminearum sensu stricto (s.s.)*, NIV to *F. meridionale*, and NIV or 3-ADON to *F. cortaderiae* and *F. austroamericanum* (Tralamazza et al., 2016), all belonging to the FGSC group. All *F. graminearum s.s.* isolates were characterized as 15-ADON, while all *F. meridionale* isolates were NIV. The *F. cortaderiae* and *F. austroamericanum* fungal isolates were found to be 3-ADON, but mainly of the NIV genotype (**Table 1**).

**Table 1:** Frequency of trichothecene genotypes in members of the *Fusarium graminearum* species complex isolated from Brazilian wheat.

Species	Genotype (%)		
*n* = 100	15-ADON	3-ADON	NIV
*F. graminearum s.s.*	43	0	0
*F. meridionale*	0	0	46
*F. cortaderiae*	0	1	7
*F. austroamericanum*	0	1	2
Total	43	2	55

Regarding the origin of the fungal isolates, there were more NIV producers among strains from SP and RS, while the 15-ADON profile was more predominant among strains from PR. The 3-ADON genotype was not found in strains from RS (**Table 2**). Despite variations in frequency, the presence of the 15-ADON and NIV genotypes was constant in all three regions.

**Table 2:** Frequency of trichothecene genotypes in members of the *Fusarium graminearum* species complex isolated in different wheat-producing regions of Brazil.

Region	Genotype (%)		
*n* = 100	15-ADON	3-ADON	NIV
PR	21	1	12
SP	10	1	24
RS	12	0	19

### Trichothecene Genotype Identification by Quantitative PCR

The results showed that all wheat samples were contaminated with fungi carrying the 15-ADON genotype. The NIV and 3-ADON profiles varied according to region (**Table 3**). In SP, PR, and RS, the NIV genotype was detected in 68, 72, and 100% of the samples, respectively. The frequency of the 3-ADON genotype was lower and this genotype was found in 12, 36, and 52% of the samples from SP, PR, and RS, respectively.

**Table 3:** Frequency of trichothecene genotypes in wheat grains from different regions of Brazil.

	Frequency (%)		
Region	15-ADON	3-ADON	NIV
SP	100	12	68
RS	100	52	100
PR	100	36	72

**FIGURE 1:**
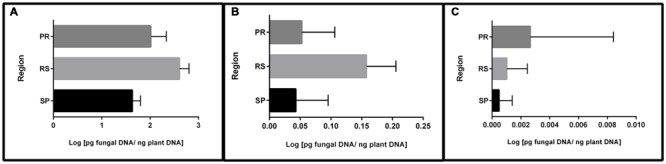
**Mean DNA concentration of trichothecene genotypes in wheat grains from different regions of Brazil. (A)** 15-ADON; **(B)** NIV; **(C)** 3-ADON. Bars indicate the standard deviation.

In addition to frequency, qPCR permitted the quantification of fungal DNA. The results showed that most of the quantified DNA belonged to fungi carrying the 15-ADON genotype (96%), followed by a small portion of the NIV genotype (3.4%) and a non-significant quantity of the 3-ADON genotype (0.06%) (data not shown). Furthermore, significant DNA differences were observed between the regions studied (**Figure 1**). Samples from RS contained the highest concentrations of 15-ADON and NIV DNA, followed by samples from PR and SP. A different trend was observed for the 3-ADON genotype. Samples from PR were more contaminated than samples from RS. Nevertheless, the average amount of DNA was very low in all regions.

**FIGURE 2:**
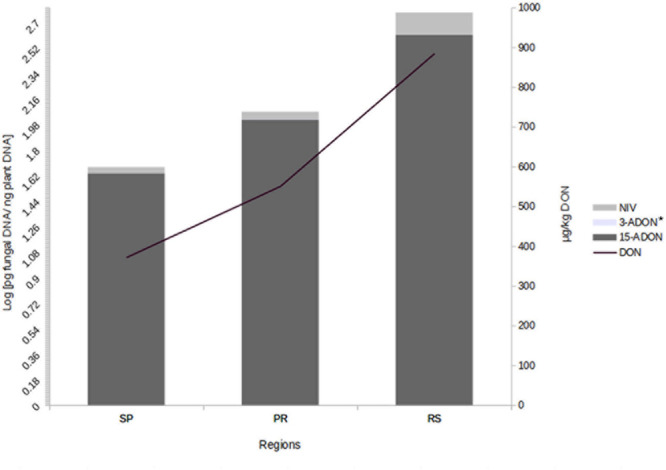
**Mean DNA concentration of trichothecene genotypes and deoxynivalenol (DON) in wheat grains from different regions of Brazil.** ^∗^3-ADON levels are not seen due to low concentration (Levels are: SP – 0.004, PR – 0.0026, RS – 0.001 pg fungal DNA/ng plant DNA).

**FIGURE 3:**
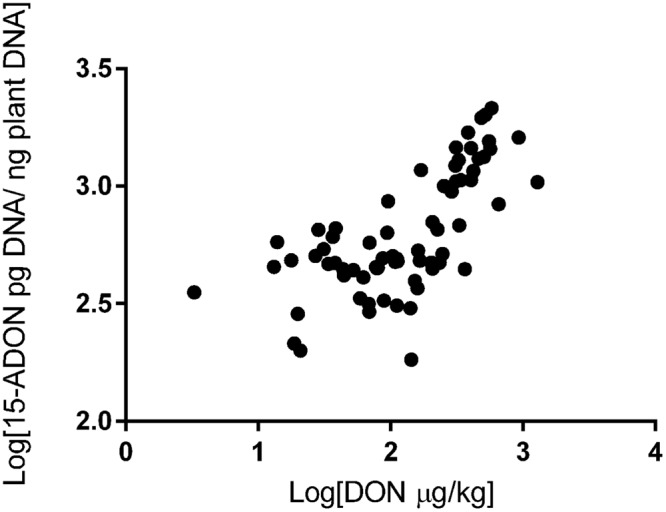
**Correlation between 15-ADON genotype DNA concentration and deoxynivalenol (DON) in Brazilian wheat grains**.

We also investigated the correlation between trichothecene genotype DNA and DON content in the wheat grains. The DON content in the wheat samples was determined in a previous study (Tralamazza et al., 2016). All 75 samples analyzed were contaminated. DON levels ranged from 183 to 1,903 μg/kg and varied across regions. The highest contamination was observed in RS (mean of 885 μg/kg), followed by PR (mean of 551 μg/kg) and SP (mean of 372 μg/kg) (data not shown). Our data showed a strong relationship between fungal DNA contamination and DON levels in the wheat samples. Grains from RS were the most contaminated by fungal DNA and showed the highest levels of DON, the same trend is seen with the samples from SP and PR (**Figure 2**). Pearson’s correlation analysis with 15-ADON + 3-ADON genotype DNA and only with 15-ADON genotype DNA showed a positive and significant correlation (*r* = 0.68, *p* < 0.001, CI 0.53–0.79) between 15-ADON genotype DNA and DON content in wheat grains (**Figure 3**). Analysis also revealed that the small concentration of 3-ADON genotype DNA did not interfere with the correlation results.

## Discussion

Two methods were used to investigate the trichothecene genotype profile of Brazilian wheat grains. Both techniques demonstrated that 3-ADON is not a relevant genotype in Brazilian wheat grains. Although detected in some wheat samples, a very small portion of DNA (0.06%) was attributed to 3-ADON fungi and only two isolates were classified as 3-ADON producers.

Almost half the fungal isolates from the wheat samples were *F. graminearum s.s.* and carried the 15-ADON genotype. DNA analysis by qPCR revealed that the vast majority of the quantified DNA was from 15-ADON producers. Our results indicated that fungal DNA contamination had an impact in the DON levels content at each region studied. Also, analysis exhibited a significant correlation between 15-ADON DNA concentration and DON content in wheat grains. Other studies have reported a positive correlation between fungal biomass and DON using qPCR. Nielsen et al. (2012), working with wheat grains, found a strong correlation between DON and *F. graminearum* and between NIV and *F. culmorum*. Similar results have been reported in other studies correlating DON and *F. graminearum* biomass (Demeke et al., 2010; Horevaj et al., 2011).

Discrepant results were observed regarding the NIV genotype. More than half the fungal isolates carried the NIV genotype and were identified as *F. meridionale*, indicating a possible role of this species as a causative agent of FHB. However, different results were obtained when the wheat grains were analyzed by qPCR. The results showed the presence of NIV producers in most wheat samples analyzed, but the DNA concentration of fungi carrying the NIV genotype was proportionally smaller than that of the 15-ADON genotype.

Trichothecene genotype profiles have been reported for Brazilian wheat, but all studies have focused on fungal isolation analysis. Previous studies have reported *F. graminearum s.s.* (15-ADON) as the most frequent profile in wheat grains (93–83%), followed by small quantities of the NIV genotype (7–13%), and no or <1% contamination with 3-ADON fungi (Scoz et al., 2009; Astolfi et al., 2012; Del Ponte et al., 2015). To our knowledge, this is the first study to identify and quantify fungal genotypes directly in Brazilian wheat grains. The result of DNA quantification and the positive relationship with DON supports the role of *F. graminearum s.s.* (15-ADON) as the main FGSC species and strongly suggests that this species is responsible for the majority of DON production in Brazilian wheat grains in all three regions studied.

In the present study, strains carrying the NIV genotype were the most frequently isolated fungi in the wheat samples. QPCR analysis of the samples indicated that, although present in most samples, less than 4% of the target DNA belonged to NIV fungi. We could not explain why several *F. meridionale* strains were isolated, although 96% of the DNA detected in the samples was 15-ADON DNA. However, we hypothesize that the conditions of incubation and grain selection might have opened an opportunity for the emergence of other fungi.

Although the qPCR findings could be affected by DNA from other trichothecene-producing *Fusarium* species (e.g., *F. culmorum*, an NIV producer), the fungal isolation data together with existing studies on Brazilian *Fusarium* wheat mycobiota and genotype strongly indicate that most relevant species belong to the FGSC complex, especially those described in our study. Thus, even if present, it is unlikely that other trichothecene-producing species have significantly interfered with the results.

In the present study, a larger number of NIV genotype fungi were isolated than previously reported (Scoz et al., 2009; Astolfi et al., 2012; Del Ponte et al., 2015). Grain selection may have contributed to the differences found. The studies cited used FHB damaged kernels, while our wheat grains showed no signs or symptoms of FHB. In FHB damaged kernels, the tissue is heavily infected with the plant pathogen, a condition that decreases the chance of emergence of other fungi. Xu et al. (2007), studying the infection of wheat with different FHB pathogens (*F. graminearum, F. poae, F. culmorum*, and *F. avenaceum*), showed that the combination of pathogens led to competition between these species and to a substantial reduction in fungal biomass (>90% reduction) of the weaker pathogen.

Another possible explanation for the findings is that, under crop conditions, *F. graminearum* may be fitter than *F. meridionale* to infect and spread in plants, as suggested by some studies (Goswami and Kistler, 2005; Spolti et al., 2012). *In vitro*, fungal isolation at controlled temperature and on different substrates (PDA medium) may have interfered with factors such as fitness, aggression and growth rate and yielded the unexpected result. Nevertheless, the data should be viewed with caution. Although only 3.4% of the fungal biomass quantified was attributed to the NIV genotype, almost all wheat samples were to some extent positive for the NIV genotype. Del Ponte et al. (2012) detected NIV in 54/66 wheat grain samples from RS (mean level of 337 μg/kg), indicating a possible role of NIV fungi as a plant pathogen and mycotoxin producer in Brazilian wheat. Reports currently show *F. graminearum* (15-ADON) as the main causative agent of FHB and DON production in Brazilian wheat; however, it seems prudent to monitor the possible introduction of new genotypes or shifts.

In North America, the occurrence of 3-ADON strains has been increasing over the last decade, replacing the formerly predominant 15-ADON genotype as the main profile in wheat crop (Ward et al., 2008; Schmale et al., 2011). Some studies speculate that 3-ADON fungi are more aggressive and produce more DON than 15-ADON fungi (Puri and Zhong, 2010). Similar results were obtained when 3-ADON and NIV fungi were compared. In contrast, other authors did not find significant differences in aggressiveness or trichothecene production between fungi carrying the 3-ADON and 15-ADON genotypes (Spolti et al., 2012). The reason for this shift remains unclear. In Uruguay, *F. graminearum s.s.* (15-ADON genotype) is also the predominant FHB agent in wheat crop, but high levels of *Fusarium asiaticum* (NIV genotype) have been identified in new wheat crop areas near rice plantations (Umpiérrez-Failache et al., 2013). Rice crop has been reported to be highly infected with *F. asiaticum* (NIV genotype) in Brazil (Gomes et al., 2015).

For a better understanding of disease symptoms and mycotoxin production, it is essential to determine the potential of the main fungal pathogens in cereal grains. Comparison of fungal isolation and conventional PCR with wheat DNA analysis by qPCR showed that both methods clearly made important contributions to the data obtained. Choosing two approaches permitted us to widen the perspective on the subject. The fungal isolation from the wheat samples provided a variety of isolated species and the possibility to work with the pathogen itself. In contrast, the use of qPCR gave us new information about trichothecene profiles in Brazilian wheat and showed for the first time a direct correlation between DON content and 15-ADON DNA levels, as well as high contamination with 15-ADON isolates in the grains, supporting the evidence that *F. graminearum s.s*. (15-ADON) is the main causative agent of FHB and producer of DON in Brazilian wheat. Although, the technique is more expensive and requires a higher technical level, the use of qPCR provided us with new information regarding the trichothecene-producing *Fusarium* species impact, and in the future, we intend to study the correlation between genotype profiles and others types of trichothecenes to further understand the effect of different genotypes in Brazilian wheat crops.

## Author Contributions

Conceived and designed the experiments: ST, RB, and BC. Performed the experiments: ST and RB. Analyzed the data: ST, RB, and BC. Wrote the paper: ST. Revised the paper: RB and BC.

## Conflict of Interest Statement

The authors declare that the research was conducted in the absence of any commercial or financial relationships that could be construed as a potential conflict of interest.
